# Modeling the Control of Trypanosomiasis Using Trypanocides or Insecticide-Treated Livestock

**DOI:** 10.1371/journal.pntd.0001615

**Published:** 2012-05-15

**Authors:** John W. Hargrove, Rachid Ouifki, Damian Kajunguri, Glyn A. Vale, Stephen J. Torr

**Affiliations:** 1 DST/NRF Centre of Excellence in Epidemiological Modelling and Analysis (SACEMA), University of Stellenbosch, Stellenbosch, South Africa; 2 Natural Resources Institute, University of Greenwich, Greenwich, United Kingdom; IRD/CIRDES, Burkina Faso

## Abstract

**Background:**

In Uganda, Rhodesian sleeping sickness, caused by *Trypanosoma brucei rhodesiense*, and animal trypanosomiasis caused by *T. vivax* and *T. congolense*, are being controlled by treating cattle with trypanocides and/or insecticides. We used a mathematical model to identify treatment coverages required to break transmission when host populations consisted of various proportions of wild and domestic mammals, and reptiles.

**Methodology/Principal Findings:**

An *R*o model for trypanosomiasis was generalized to allow tsetse to feed off multiple host species. Assuming populations of cattle and humans only, pre-intervention *R*o values for *T. vivax*, *T. congolense*, and *T. brucei* were 388, 64 and 3, respectively. Treating cattle with trypanocides reduced *R*_0_ for *T. brucei* to <1 if >65% of cattle were treated, *vs* 100% coverage necessary for *T. vivax* and *T. congolense*. The presence of wild mammalian hosts increased the coverage required and made control of *T. vivax* and *T. congolense* impossible. When tsetse fed only on cattle or humans, *R*_0_ for *T. brucei* was <1 if 20% of cattle were treated with insecticide, compared to 55% for *T. congolense*. If wild mammalian hosts were also present, control of the two species was impossible if proportions of non-human bloodmeals from cattle were <40% or <70%, respectively. *R*_0_ was <1 for *T. vivax* only when insecticide treatment led to reductions in the tsetse population. Under such circumstances *R*_0_<1 for *T. brucei* and *T. congolense* if cattle make up 30% and 55%, respectively of the non-human tsetse bloodmeals, as long as all cattle are treated with insecticide.

**Conclusions/Significance:**

In settled areas of Uganda with few wild hosts, control of Rhodesian sleeping sickness is likely to be much more effectively controlled by treating cattle with insecticide than with trypanocides.

## Author Summary

In Uganda, cattle are an important reservoir for *Trypanosoma brucei rhodesiense*, the causative agent of Rhodesian sleeping sickness (human African trypanosomiasis), transmitted by tsetse flies *Glossina fuscipes fuscipes*, which feed on cattle, humans, and wild vertebrates, particularly monitor lizards. Trypanosomiasis can be controlled by treating livestock with trypanocides or insecticide – killing parasites or vectors, respectively. Mathematical modeling of trypanosomiasis was used to compare the impact of drug- and insecticide-based interventions on *R*_0_ with varying densities of cattle, humans and wild hosts. Intervention impact changes with the number of cattle treated and the proportion of bloodmeals tsetse take from cattle. *R*_0_ was always reduced more by treating cattle with insecticide rather than trypanocides. In the absence of wild hosts, the model suggests that control of sleeping sickness (*R*_0_<1) could be achieved by treating ∼65% of cattle with trypanocides or ∼20% with insecticide. Required coverage increases as wild mammals provide increasing proportion of tsetse bloodmeals: if 60% of non-human bloodmeals are from wild hosts then all cattle have to be treated with insecticide. Conversely, it is reduced if lizards, which do not harbor trypanosomes, are important hosts and/or if insecticides are used at a scale where tsetse numbers decline.

## Introduction

Across sub-Saharan Africa, a variety of *Trypanosoma* spp transmitted by tsetse flies (*Glossina* spp) cause human and animal trypanosomiases. There are >10,000 cases/year of Human African Trypanosomiasis (HAT) [1] with an estimated burden of ∼1.3 million Disability Adjusted Life Years (DALYs) [2] and economic losses in excess of $1 billion due to human and animal trypanosomiasis [3]. While interventions can be directed against the vector or the parasite, emphasis has usually been on the use of drugs to treat the disease both in humans and in livestock.

While the importance of treating cases, especially human ones, cannot be overstated, several advances in our understanding of tsetse biology and ecology, and improvements in the cost-effectiveness of tsetse control [4], [5], have revived interest in that approach to disease management. First, the use of satellite navigation as an aid to nocturnal aerial spraying, spraying much larger areas than previously, and protecting the sprayed areas with odor-baited targets, has provided impressive results, such as the eradication of *G. m. centralis* from Botswana [6]. Second, the demonstration of the importance of odor for host location in some species of tsetse provided a means of attracting them to insecticide-treated targets and, by killing the flies, provided control of cattle and human trypanosomiasis [7]–[10]. Third, the particularly low reproductive rate in tsetse made it possible to use as few as four such targets per square kilometer to eliminate isolated populations of *G. pallidipes* Austen and two sub-species of *G. morsitans* [9], [11]. The method is cheaper than aerial spraying and more environmentally friendly than insecticidal ground spraying, game destruction or habitat clearance [11]. Issues of cost, logistics, government commitment, and theft of materials have meant, however, that the approach has not been used in large-scale control programs except in Zimbabwe and in the Western Province of Zambia [11], [12].

Part of the reason for this limited use stems from the fact that, simultaneously with the development of insecticide-treated target technology, it was realized that tsetse control could be achieved equally effectively by applying insecticide to the very livestock - generally cattle - off which the tsetse were feeding. This approach has been used very successfully in areas where tsetse feed predominantly on cattle [13], [14], though it would be less effective in areas where – as in large parts of Zimbabwe and Tanzania – the predominant food source for the tsetse are wild mammals.

Whereas insecticide-treated cattle (ITC) can be used in operations aimed at eliminating tsetse populations, animal trypanosomiasis can also be reduced to low levels even where tsetse populations persist [15]. It is, of course, relief from cattle disease – rather than issues of tsetse fly control versus eradication – which most interests stockholders in tsetse areas and which can be used to interest the stockholder in becoming actively involved in tsetse and trypanosomiasis control [13]. Recent advances in our understanding of the feeding behavior of tsetse on cattle have led to even cheaper methods of tsetse control where the insecticide is applied to the body regions and/or individual animals on which most tsetse feed [16], [17]. This restricted application of pyrethroids is comparable in its cost and simplicity to the widespread use of trypanocides by farmers to prevent or cure trypanosomiasis in their livestock [16].

There are several possible reasons why these advances in affordable, low-technology tsetse control have not, as yet, played a significant role in efforts against HAT. First, there is an imperative to find and treat infected humans and livestock and this approach is thus the foundation of all efforts against the disease. Second, the odor-baited devices used so effectively in efforts against animal trypanosomiasis [10] are less effective against the important vectors of HAT [18], [19]. This poor efficacy is probably related, in part, to the distinctions between the host relationships of the various tsetse species. The important vectors of animal trypanosomiasis, *i.e.*, the Morsitans-group tsetse, feed almost exclusively on mammals (*e.g.* warthog, kudu, buffalo and cattle) which they locate largely by odor, whereas the Palpalis-group species, which are the main vectors of HAT, are less responsive to odors and include reptiles and birds in their diet. For instance, between 50 and 90% of meals taken by *Glossina fuscipes fuscipes* are from monitor lizard [20] which themselves do not support all the trypanosome species infective to mammals [21].

In this paper, we investigate the theoretical effects of two different approaches to trypanosomiasis control, both of which have already been shown to be of interest to small-scale stockholders in resource-limited settings [22]. First we consider the effect of treating animals with trypanocides, which prevent the disease without having any insecticidal effect. Second, we consider the use of the ITC method, which has no direct trypanocidal effect but which increases mortality in the vectors. We limit our study to the situation typical of eastern and southern Africa, where *Trypanosoma vivax*, *T. congolense* and *T. brucei rhodesiense* occur in livestock and wildlife - and where the last-named parasite also causes “Rhodesian” sleeping sickness in humans [23], [24].

## Methods

We generalize the Rogers [25] two-host model for trypanosomiasis to one where a single species of tsetse can feed off any finite number (*n*) of vertebrate hosts. The formal proof that Rogers' model can be generalized in this way is given in the Supporting Information (Text S1). The overall basic reproductive rate (*R*_0_) of a trypanosome species is given by:
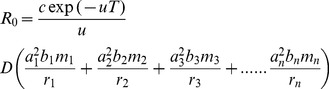
(1)where *D* = 1 for *T. vivax* and *T. congolense* and
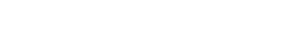
for *T. brucei*, and where the following definitions apply: *R*_0_ = overall basic reproductive rate; formally, in a completely susceptible population, the number of trypanosome-infected tsetse arising from each infected fly; *c* = P(infected blood meal gives mature infection in fly); *u* = Daily mortality rate of the flies; *T* = Incubation period in tsetse (all time units are days); *a_i_* = *p_i_*/*d*, where *p_i_* = Proportion of tsetse bloodmeals from species *i*, *d* = Duration of feeding cycle in flies; *b_i_* = P(infected fly bite produces infection in species *i*); *m_i_* = *V*/*N_i_*, where *V* = Number of tsetse, *N_i_* = Number of animals of species *i*, 1/*r_i_* = Duration of infection in species *i*. The parameter *D* differs between *T. brucei* and the other species of trypanosomiasis because it is assumed that tsetse can only be infected with *T. brucei* when they take their first bloodmeal. It is assumed that the probability of infection for the other species is independent of a fly's feeding history: to distinguish this situation Rogers also replaced *c* with *c*′ for *T. brucei* [23]. The default values for the parameters of his two-host model for Rhodesian sleeping sickness [23] are copied here for convenience, in Tables 1 and 2.

**Table 1:** General default variable and parameter values for the Rogers model for two-host species trypanosomiasis [25].

		Default
*N_1_*	Number of animals of species 1	300
*N_2_*	Number of animals of species 2	50
*V*	Number of tsetse	5000
*p*_1_	Proportion of tsetse bloodmeals from species 1	0.3
*P*_2_	Proportion of tsetse bloodmeals from species 2	0.7
*u*	Daily mortality rate of the flies	0.030
*d*	Duration of feeding cycle in flies	4
	*a_1_* = *p_1_*/*d*, *a_2_* = *p_2_*/*d*, *m_1_* = *V*/*N_1_*, *m_2_* = *V*/*N_2_*	

See the original paper for further details and Table 2 for default parameter values specific for the different species of trypanosomes.

**Table 2:** Default parameter values for the Rogers (1988) model for two-host species trypanosomiasis: values specific for the different species of trypanosomes.

		*T. vivax*	*T. congolense*	*T. brucei*
1/*i*_1_	Incubation period in species 1	-	-	12
1/*i*_2_	Incubation period in species 2	12	15	12
1/*r*_1_	Duration of infection in species 1	-	-	70
1/*r*_2_	Duration of infection in species 2	100	100	50
1/*v*_1_	Duration of immunity in species 1	-	-	50
1/*v*_2_	Duration of immunity in species 1	100	100	50
*T*	Incubation period in tsetse	10	20	25
*b*_1_	P(infected fly bite produces infection in species 1)	-	-	0.62
*b*_2_	P(infected fly bite produces infection in species 2)	0.29	0.46	0.62
*c*	P(infected blood meal gives mature infection in fly)	0.177	0.025	0.065
*c*′	P(infected blood meal gives mature infection in fly)	-	-	0.065

We extend the model to consider cases where, in addition to humans and domestic stock (cattle), the following vertebrate species are present: (1) wild mammals; (2) monitor lizards; (3) wild mammals and monitor lizards. The interventions to be considered involve the treatment of cattle with: (1) prophylactic trypanocides that kill trypanosomes but have no effect on tsetse mortality; (2) ITC, *i.e.*, topical application to hosts of insecticides that kill tsetse but have no direct effect on trypanosome mortality.

The use of ITC can reduce *R*_0_ in two ways. First, in common with all insecticidal techniques, it reduces the average life expectancy of tsetse, so decreasing the abundance of the flies and the proportion of the population that is old enough to harbor mature, transmissible infections. Second, and in contrast with other insecticidal techniques such as traps or insecticide-treated targets, ITC kills specifically those tsetse that become infected from the reservoir of disease in cattle.

Since the Rogers model assumes that the abundance and age structure of the tsetse population is constant, it is particularly suitable for highlighting the second type of effect, and so for comparing ITC and trypanocides as means of reducing the probability that a fly will become infected. In the present paper we first use the Rogers model to address this matter under circumstances in which various levels of the use of trypanocides or insecticide treatment are applied to cattle that represent different proportions of the overall cattle population, and with host populations composed of various species. We then identify the extra benefit that ITC produces via reductions in the abundance and mean age of the tsetse population, and predict the relative merits of using ITC and trypanocides, as assessed via the model.

## Results

### Rogers model

As a preliminary check we inserted the published default parameter values (see Tables 1 and 2, [25]) into Equation (1) for the scenario where only (untreated) cattle and humans provided the source of tsetse bloodmeals, and obtained the published values for *R*_0_: 388.2 for *T. vivax*, 64.4 for *T. congolense*, and 2.65 for *T. brucei*. The last value is made up the sum of two components, 2.54 from the cattle and 0.11 from humans, implying that *T. brucei* would not survive in the absence of the cattle reservoir [25]. To control, and eventually eliminate, *T. brucei* the goal therefore must be to reduce the combined *R*_0_, for human and non-human hosts, to a value less than unity.

### Effect on R_0_ of treating a proportion of cattle with trypanocidal drugs

#### Tsetse feed off cattle and humans only

In considering the effects of this intervention note that, for simplicity and to consider the case where trypanocides could have maximum effect, we consider that a proportion of the cattle population is kept continuously on trypanocidal drugs that have 100% efficacy. We assume that flies feed at random off cattle with respect to their treatment status; but the treated cattle are never infected with trypanosomiasis and cannot pass on the infection. Effectively, therefore, we can consider the cattle which are on trypanocides as a third vertebrate species – differing only from untreated cattle in that *b_i_* = 0 so that, obviously, these animals contribute nothing to *R*_0_ – see Equation (1).

**Figure 1:**
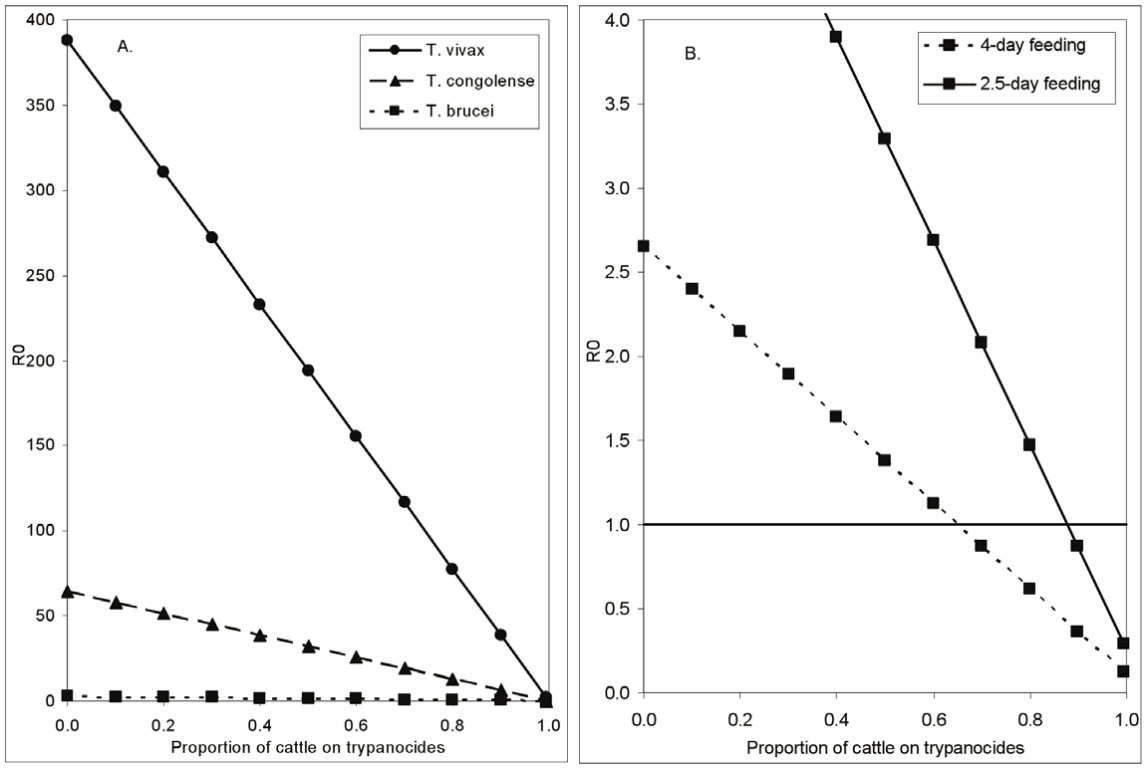
The effect on trypanosomiasis of treating cattle with trypanocides. A. The effect on the basic reproductive value, *R*_0_, for three species of trypanosome of treating a proportion of cattle with trypanocides such that the cattle have zero probability of being infected with trypanosomes of any species. B. Rescaled version showing details of change in *R*_0_ for *T. brucei* and also the effect of decreasing the tsetse feeding interval from 4 days to 2.5 days.

With the default parameter input values, *T. vivax* or *T. congolense* can be satisfactorily controlled in cattle only if 100% of the stock are kept on continuous and completely effective treatment (Figure 1A). For *T. brucei*, however, the disease could be controlled, and even eliminated, if cattle were the only source of bloodmeals other than humans. In order to achieve this, >65% of the cattle would need to be on continuous, perfectly effective, treatment with trypanocides (Figure 1B). The proportion required to be on treatment rises to about 90% if, as likely for *G. pallidipes* females [26], [27], the feeding interval is 2.5 days rather than the 4 days suggested in Table 1.

Preliminary investigations of the sensitivity of the estimated value of *R*_0_ to changes in various input parameters showed that if the cattle numbers were increased from 50 to 100 then it was necessary to have only 30% of the cattle on trypanocides to make *R*_0_<1 for *T. brucei*. Conversely, reducing the number of cattle from 50 to 10 the baseline *R*_0_ increased to 12.8 for *T. brucei*. If, also, the proportion feeding off humans was reduced to the order of 5%, *R*_0_ for this species increased to 23.4. Under these circumstances >90% of cattle must be on trypanocides for control of *T. brucei*.

These results, based on changes in only one or two parameters, need to be treated with some caution, however, because it has been assumed that the said changes can be made without effect on other parameters. In particular it has been implicitly assumed that we can change the cattle density without affecting the tsetse density, and without affecting the proportion of flies that feed off humans. More complete sensitivity analyses are, however, beyond the scope of this study and will be presented elsewhere. The above results suggest, nonetheless, that it will generally be very difficult to control human trypanosomiasis by treating cattle with prophylactic trypanocides – even when there is no additional wildlife reservoir of trypanosomes present. Note that there are no prophylactic drugs for humans, so that treatment of humans is restricted to those who are infected with trypanosomes. The contribution of humans to *R*o is anyway very small [23].

#### Tsetse feed off cattle, humans and monitor lizards

If the wild vertebrate host is a monitor lizard, which does not get infected with trypanosomiasis when bitten by an infected tsetse, the dynamics of the disease are identical to the situation where a proportion of cattle are kept permanently on perfectly effective trypanocides. In both cases a proportion of tsetse blood meals are taken from hosts that neither suffer from nor transmit trypanosomiasis. The situation is thus represented by the results in Figure 1 and we need only change the label on the abscissa, to read: ‘Proportion of non-human tsetse meals taken from lizards’.

#### Tsetse feed off cattle, humans and wild mammals

**Figure 2:**
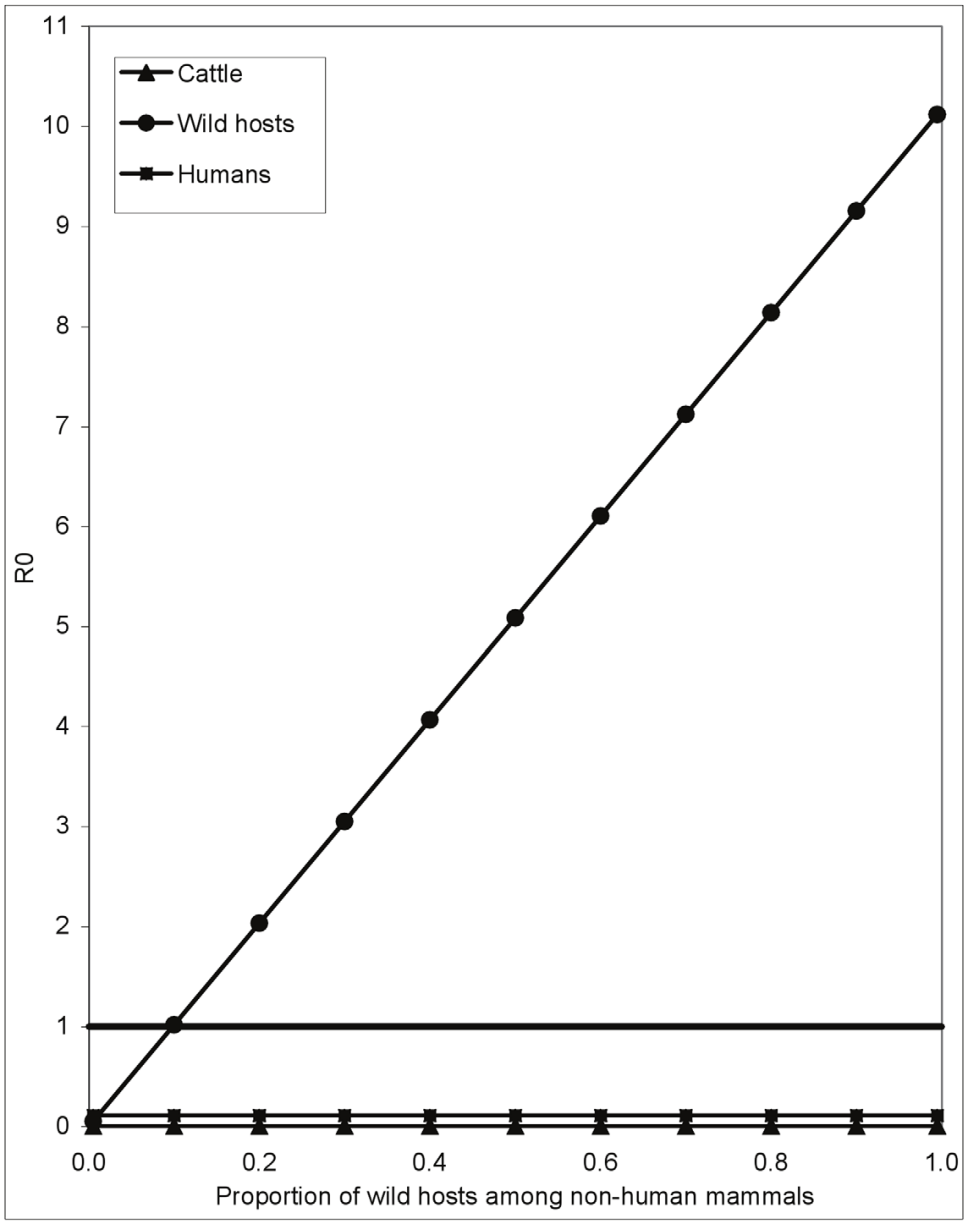
How the presence of wild hosts affects the control of *T. brucei* using trypanocides. The relationship between the proportion of wild mammals among all non-human hosts and the basic reproductive value (*R*_0_) for *T. brucei*. It is assumed that any cattle present are all, continuously, on a trypanocide with 100% efficacy.

When cattle are kept in areas where there is wildlife that, by assumption, cannot be treated with trypanocides, it will be even more difficult to reduce *R*_0_ below unity. In fact, as long as wild mammalian hosts form >10% of the tsetse diet, the contribution to *R*_0_ for *T. brucei* from the wild mammal component of the host population is always >1 (Figure 2) so that, even when 100% of the cattle population are treated with trypanocides, the disease cannot be eradicated. The same, naturally, also applies to *T. vivax* and *T. congolense*. Notice that the *R*_0_ for *T. brucei* in cattle is always zero because we assume here that any cattle that are present are constantly on totally effective trypanocide treatment. *R*_0_≈10 for *T. brucei* when there are only wildlife present (Figure 2) whereas, when the same number of untreated cattle are present, *R*_0_<3 (Figure 1B). This is due to the fact that we are assuming that wildlife do recover from a trypanocide infection. The *R*_0_ for humans is not zero, but is very low, with the disease is now present in humans due solely to the reservoir in (untreated) game animals.

It appears from the above results that, whereas trypanocides can, and have been, used to provide short-term protection against trypanosomiasis, they will never provide a long-term solution to the disease in cattle. Moreover, it will be difficult to use the mass treatment of reservoir hosts (i.e., cattle) with trypanocides to control *T. brucei* in humans. The underlying problem is simply that the trypanocides have no effect on the mortality, abundance and age structure of the vector population.

### Effect on R_0_ of treating a proportion of cattle with insecticide

We now turn to the use of the insecticide-treated cattle (ITC) method of control – where the vectors, rather than the trypanosome, are targeted. In the previous sections we have assumed a fixed daily rate for adult tsetse mortality (Table 1). When considering the use of ITC, however, we need to decompose this factor into the mortality occurring at the time of feeding and that occurring between feeds. The former has generally been considered the dominant component [28], [29] even where the host is not treated with insecticide.

If the probability of surviving a feed is *q_f_* and the probability of surviving a non-feeding day is *q_n_* then a fly survives a complete feeding cycle of *d* days with probability *q_f_ q_n_^d^*. With *q_f_* = 0.96, *q_n_* = 0.98, and with the assumed four-day feeding interval [25], the probability of surviving from one feeding cycle would then be approximately 0.96×0.98^4^ = 0.885 and the daily mortality rate is calculated as −ln(0.885)/4≈0.03, as originally assumed [25].

Where some hosts are treated with insecticide we assume that flies always die if they feed off a treated animal; the probability of a given fly surviving a feed is thus the product of the probabilities that it feeds off an un-treated host and survives that meal. We assume further that flies feed off all cattle at random, particularly with respect to the animal's treatment status. If the proportion of cattle treated is *p_i_* then the probability of a fly surviving a feeding cycle is now (1−*p_i_*) *q_f_ q_n_^d^*. For example, with the above values for *q_f_*, *q_n_* and *d*, and if 10% of the cattle are treated, the survival probability will be 0.9×0.885 = 0.797 and the daily mortality is now approximately 0.057. As a first approximation we ignore any extra mortality arising from a fly feeding off a human, rather than cattle or wildlife.

#### Tsetse feed off cattle and humans only

**Figure 3:**
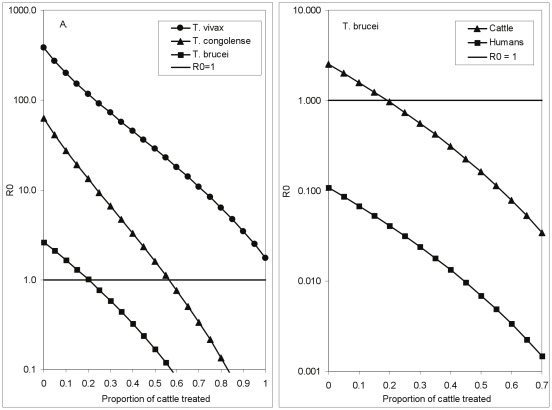
The effect on trypanosomiasis of treating cattle with insecticide. A. The effect on the basic reproductive value, *R*_0_, for three species of trypanosome of treating a proportion of cattle with insecticide that kills any fly attempting to feed off a treated animal. B. Detail for *T. brucei*, showing the differential effects on *R*_0_ for cattle and for humans.

It is immediately evident that our model suggests the use of ITC provides a much more promising prospect than trypanocides for trypanosomiasis control. In a situation where the only tsetse hosts are cattle and humans it is only necessary to treat about 20% of the cattle with insecticide in order for the *R*_0_ for *T. brucei* to fall below unity (Figure 3A). Moreover, whereas it was essentially impossible to use trypanocides to make *R*_0_<1 for *T. congolense*, this end can be achieved with ITC if about 55% of cattle are treated. For *T. vivax*, however, *R*_0_>1 even when all cattle are treated with insecticide (Figure 3A). This last result may appear counter-intuitive until it is recalled that we are assuming that the tsetse population is constant, regardless of the proportion of cattle treated with insecticide. This could happen if cattle are treated over a small area, surrounded by large areas with abundant flies feeding off untreated hosts. Under such circumstances natural birth within the treated area, and invasion from outside it, could be sufficient to maintain the tsetse population at a constant level and, thereby, *R*_0_>1 for the more rapidly reproducing *T. vivax*.

Changing the feeding frequency has a smaller effect on *R*_0_ than in the case of trypanocide treatment, because increased feeding rates increase both the probability of infection and of the fly dying during the feeding process and these two increases have effects on *R*_0_ that tend to cancel each other out. Similar antagonistic effects on *R*_0_ arise from the fact that flies visit, and probe, a host animal more than once before obtaining a bloodmeal [17], [30].

#### Tsetse feed off cattle, humans and wildlife

**Figure 4:**
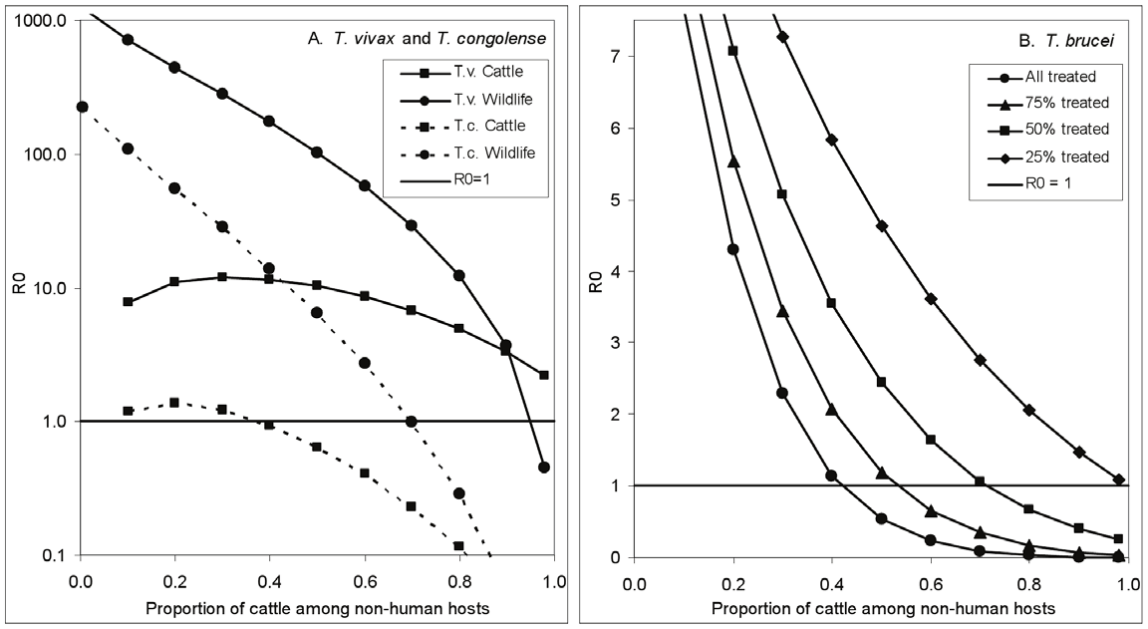
How the presence of wild hosts affects the control of *T. brucei* using insecticide. The relationship between the basic reproductive value, *R*_0_, and the proportion of cattle among all non-human mammalian hosts in the situation where a proportion of cattle are treated with insecticide that kills any fly attempting to feed off that animal. A. *T. vivax* and *T. congolense* in cattle and wildlife - where all cattle are treated with insecticide. B. *T. brucei*; total *R*_0_ in humans, cattle and wildlife for situations where different proportions of the cattle are treated with insecticide.

Where tsetse can feed off wild mammals or reptiles which cannot be treated with insecticide it will, of course, be more difficult to control trypanosomiasis using ITC. Calculations similar to those above can be used to estimate the probability of a fly surviving a feeding cycle. As is obvious from Figure 3, even when all cattle are always treated with an insecticide of 100% efficacy, the *R*_0_ for *T. vivax* for cattle and wildlife combined cannot be reduced to <1 – under the assumption that the tsetse population is constant (Figure 4A). For *T. congolense* in cattle the proportion of cattle among non-human hosts would have to be >40% for ITC to be able to force *R*_0_<1 (Figure 4A). Even at that level, however, *R*_0_ in wildlife would still be >10 and would provide a constant source of re-infection. To make *R*_0_<1 for *T. congolense* in cattle and wildlife combined >70% of the non-human bloodmeals would need to be taken from cattle. Cattle would need to comprise ∼40% of non-human tsetse bloodmeals for *R*_0_<1 in *T. brucei* – even if all cattle are treated (Figure 4B).

In reality it will be difficult to keep all cattle effectively treated all of the time; the effectiveness of the insecticide decreases with time [16] and re-treatment may not be possible at optimal intervals. These different effects will be essentially equivalent to a situation where reduced proportions of cattle are treated. Figure 4B shows how, if human trypanosomiasis is to be controlled, the required proportion of cattle among non-human hosts increases as the proportion of cattle treated decreases. Thus if only 50% of the cattle are treated, cattle must comprise at least 70% of the non-human hosts; and this figure rises to 100% if only 25% of the cattle are treated. Control of *T. vivax* and *T. congolense* would be correspondingly more difficult under these circumstances.

### Effect of insecticide-treated cattle on tsetse population

Figures 1, 2, 3, and 4 provide estimates of the control of trypanosomiasis, by way either of the use of trypanocidal drugs or ITC, in the situation where there is sufficient birth, of uninfected flies, to ensure that the tsetse population stays at a constant level [25]. This should be a reasonable assumption in the case where trypanocidal treatment is used to control trypanosomiasis and there is no imposed mortality on the tsetse population.

When ITC is used, the population could only be kept constant if the increase in mortality is balanced by an increase in birth and/or immigration. If birth is the predominant source of replacements then Figures 3 and 4 reflect the control situation. If, however, the population is kept constant due to immigration then the replacement flies will be predominantly older flies, with above-average probability of being infected with trypanosomes, so that Figures 3 and 4 over-estimate the efficacy of ITC.

However, where ITC is used, either against closed populations of tsetse or on a sufficiently large scale that immigration is limited at sites far from the boundary, the expectation is that the fly population will decrease. Inspection of Equation (1) shows that, other things being equal, *R*_0_ changes linearly with the tsetse population so that, where the use of ITC produces a decline in population levels the effect on *R*_0_ will be larger than indicated in Figure 3. We follow Smith & McKenzie [31] in estimating that, if mortality was increased from some value *u* to *u*′, the initial vector population (*V*) would decrease to *Vu*/*u*′.

**Figure 5:**
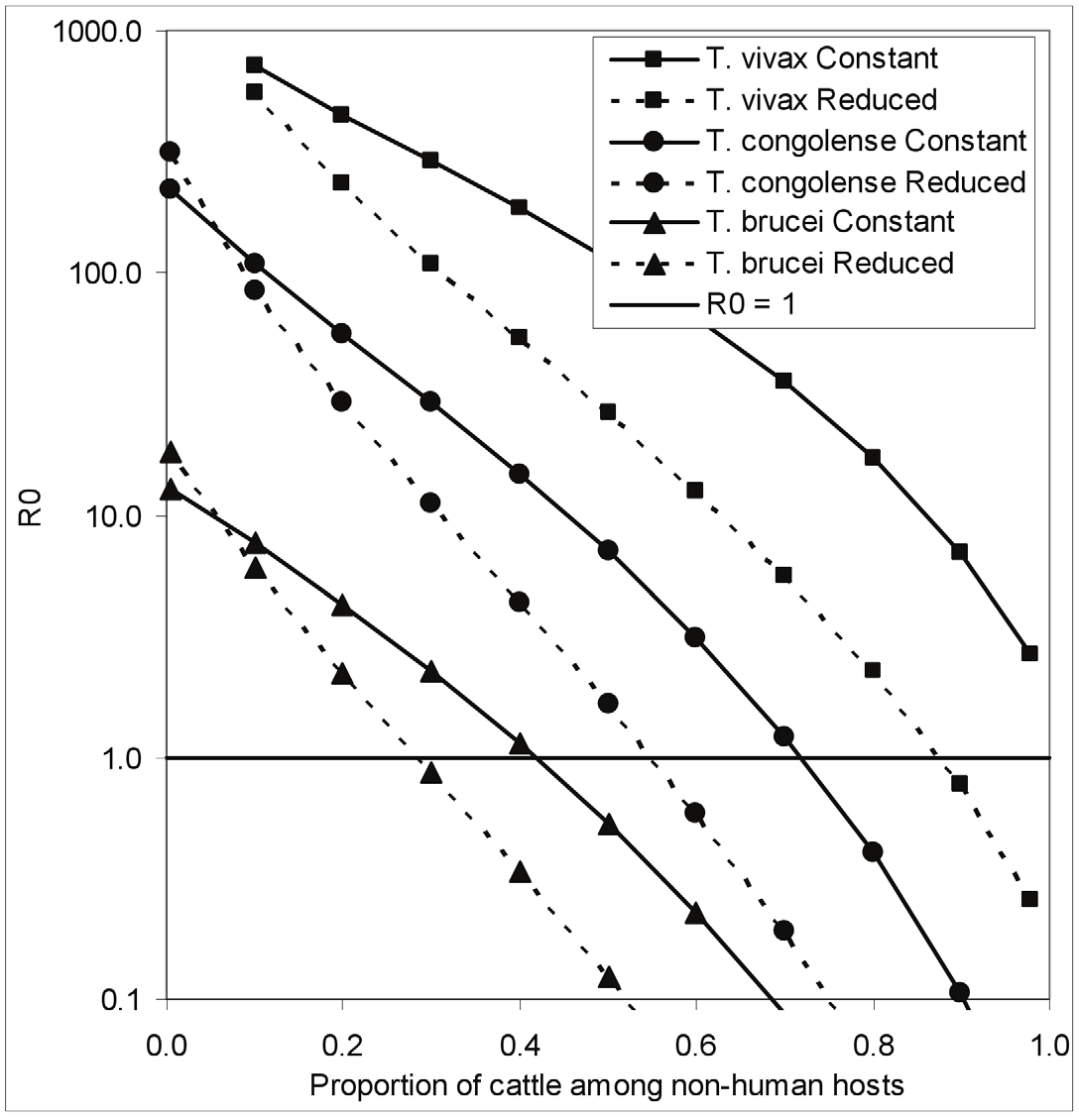
The effect of vector population reduction on the control of *T. brucei* using insecticide. The relationship between the basic reproductive value, *R*_0_, and the proportion of cattle among all non-human mammalian hosts in the situation where all cattle are treated with an insecticide that kills any fly attempting to feed off that animal. The dotted lines show the further change in *R*_0_ in the circumstance that the imposed mortality results in a decline in the tsetse population.

Taking this factor into account changes the threshold value for the required percentage of cattle among non-human hosts. Thus, under the assumption of a constant tsetse population, it was impossible to force *R*_0_<1 for *T. vivax* (Figures 3A, 4A, 5). However, if tsetse populations are reduced as a consequence of ITC, *R*_0_<1 for *T. vivax* as long as cattle make up >90% of the non-human hosts (Figure 5). The proportions of cattle among non-human hosts, required to force *R*_0_<1, declines from roughly 70% to 55% for *T. congolense* and 40% to 30% for *T. brucei* (Figure 5).

## Discussion

For purposes of comparing our results with previous work we have, initially, adhered closely to the design, and the parameterization, of the Rogers model – which provides a useful tool for investigating the dynamics of trypanosomiasis. It is recognized, however, that some fundamental details of the model can be improved. For example, the model makes no distinction between male and female tsetse, which are known to differ with respect to longevity, mobility, infectivity and responses to baits [32], and does not allow that mortality changes as a function of age [33].

Moreover, advances in our knowledge over the past 23 years allow the selection of parameter values that better reflect the field situation. Thus, the feeding interval is certainly shorter than four days and where tsetse make more than one visit to a host per feeding cycle [30], [34] this will impact on both the probability that they transmit a trypanosome, and the probability that they are killed when they alight on an animal that has been treated with insecticide.

Most seriously, the model assumes that the abundance and age structure of the tsetse population is constant. This can be a reasonable assumption where no tsetse control efforts are in place, or when trypanosomiasis control consists simply of treating livestock with trypanocides that have no insecticidal effect. If cattle provide a substantial proportion of tsetse bloodmeals and if a significant proportion of these cattle are treated with insecticide, however, it may be expected that both the size of the population in the area under treatment, and its mean age, will tend to decline. On the other hand the model also ignores the problem of invasion from adjacent infected areas and this further complicates the estimation of the effect of ITC.

Finally, we have not modified Rogers' implicit assumption that tsetse feed at random off the individuals of a given host species. This is known not to be the case and this consideration will complicate the modeling [17]. Nonetheless, in the limit, where some individuals provide no bloodmeals at all for tsetse, they effectively do not exist from the modeling point of view. One could thus simplify the problem by considering the “effective” number of individuals in a herd – being the numbers that do provide bloodmeals. In the same way, baboons and impala – which provide almost no bloodmeals for tsetse – do not need to be considered when modeling the dynamics of trypanosomiasis.

It would not be easy to incorporate all of these details into the present model and still maintain the simplicity that allowed the model to be generalized to apply to the variety of situations considered here. The more general model can, however, be investigated using simulation models; the results of such an exercise will be reported in a separate paper.

Despite the above limitations, the theoretical development presented here suggests that the use of ITC should provide a potent tool for controlling, or even eliminating, trypanosomiasis in situations where cattle provide the majority of bloodmeals for tsetse. The dynamics of transmission ensure that the requisite proportion favoring the use of ITC depends on the species of trypanosome involved; for *T. vivax* there is little hope of eliminating the disease unless at least 90% of the tsetse bloodmeals are from cattle – and then only if insecticide treatment is such that all tsetse feeding off cattle are killed, and if the situation is such that the increased tsetse mortality results in a decline in the fly numbers. For *T. brucei* the situation is very much more favorable; even if 70% of bloodmeals are being taken from wildlife, treatment with insecticide of the cattle providing the remaining meals from non-humans allows *R*_0_ to be reduced to unity. The situation for *T. congolense* is intermediate between these extremes. By contrast, the use of trypanocides will never allow *T. vivax* and *T. congolense* to be eliminated, even where tsetse feed only on cattle – unless all animals are kept permanently on a perfect trypanocide. *T. brucei* could be controlled – but only in the absence of wildlife hosts.

The classical Rhodesian sleeping sickness foci are often associated with protected areas [35], the vectors are Morsitans-group tsetse and the hosts are wild mammals such as warthog, buffalo and bushbuck. Tackling these foci is very difficult: block treatment of wild hosts with trypanocides is impossible and hence vector control is the only option. Moreover, the flies are highly mobile [36] and widely dispersed across a range of habitats and hence, to be effective, tsetse control must be applied across the entire protected area. This approach is illustrated by the use of aerial spraying and insecticide-treated targets to eliminate tsetse from the Okavango Delta (area≈15,000 km^2^) of Botswana [6]. Few countries have the resources for such large-scale interventions and hence sleeping sickness persists in parts of east and southern Africa.

By contrast, tackling Rhodesian sleeping sickness transmitted by *G. fuscipes* might be more tractable for several reasons. First, the underlying *R_0_* of *T. b. rhodesiense* is likely to be low. Studies of the diet of *G. f. fuscipes* in Uganda and Kenya have shown that monitor lizards (*Varanus nilotica*) provide between ∼50% and >90% of bloodmeals [20], [37]–[39] and it seems likely that poikilothermic hosts such as monitor lizards will not be competent hosts for mammalian trypanosomes. The only published study [21] confirms this for *T. congolense* and the results for *T. brucei* are equivocal but not compelling: no human-infective trypanosome has been recovered from a lizard, only one wild lizard (N = 46) has been found with *T. brucei s.l.*, and experimental infections of captive lizards – which were not subject to the range of temperatures found in nature – produced, at most, a low and transient parasitaemia. Our results suggest that if lizards are indeed refractory to mammalian trypanosomes and form >80% of the diet of tsetse, then the *R_0_* for *T. b. rhodesiense* is less than 1. Hence we might expect that Rhodesian sleeping sickness will be associated with areas where lizards are not abundant such as away from the shores of Lake Victoria and/or in densely settled areas where wild hosts are absent. Consistent with this hypothesis, the current foci of Rhodesian sleeping sickness in Uganda are, paradoxically, not near the shores or islands of Lakes Victoria and Kyogu, where tsetse are abundant, but rather at sites further inland [40], [41].

In areas where lizards are not important hosts, then livestock, particularly cattle, are important [20], [38]. In the case of Uganda, the densities of cattle frequently exceed 50 head/km^2^ [42] and the degraded environment leads to relatively low densities of tsetse [38]. Increasing the host∶vector ratio reduces *R_0_*: for densities of 10 host/km^2^ and 5000 tsetse/km^2^ our model (with other parameters as in Tables 1 and 2) suggests *R_0_* = 13 for *T. brucei*; with 50 hosts and 5000 tsetse/km^2^ the value is 3, and with 50 hosts and 500 tsetse/km^2^ it is 0.3.

Second, *G. f. fuscipes* are restricted to riverine habitats and are less mobile than Morsitans species such as *G. pallidipes* [36] and hence vector control can be applied on a smaller scale, focused on riverine and lacustrine habitats.

Third, the abundance of cattle in settled areas, their importance as a host for tsetse and their need for water – and hence daily presence in the riverine and wetland habitats where *G. f. fuscipes* is concentrated – means that insecticide-treated cattle should be particularly effective baits. Hence, SE Uganda, the place where Rhodesian sleeping sickness is most serious, accounting for over half (2848/5086) of all cases across Africa [35], is probably the easiest to tackle.

Present evidence for the superior efficacy of ITC assumes greater importance due to indications over the last decade that the economy of this technique can be improved substantially, with no material loss of performance. The application of insecticide can be restricted to the legs and belly of cattle where most tsetse feed, thereby reducing the material costs of treatment by ∼90% [16]. In addition, since most tsetse feed on the larger and older animals within a herd [17], [43], only these animals need be treated, with further savings in cost. As a consequence, the annual material cost of ITC is reduced to <US$2 per beast per year [44] – comparable to the cost of a single dose of diminazene aceturate to cure trypanosomiasis. The restricted application of pyrethroids to older cattle allows young stock to be exposed to ticks and hence develop a natural immunity to tick-borne diseases [45] and reduces impact on dung fauna [46], [47] which play an important role in maintaining soil fertility and, ultimately, productive pasturage. Against these favorable indications for the usefulness of ITC there is the problem that the technique can be used only in districts where cattle occur, although modeling suggests that ITC can be effective even when cattle are distributed patchily, i.e., absent from bands of habitat up to several kilometers wide [48].

Nonetheless, for the densely-settled rural areas of central and southern Uganda where Rhodesian sleeping sickness is most acute, our findings suggest that relatively modest levels of treatment (∼20% even if tsetse numbers are not reduced by the intervention) could lead to the elimination of HAT. Hence there is the exciting prospect that an important public health benefit might arise through the private actions of livestock keepers using cheap, simple and environmentally-benign methods to control vector-borne diseases in their livestock [22].

## Supporting Information

Text S1 Formulation of *R*_0_.
(DOC)
