# Supplementary material for: Modeling the Control of Trypanosomiasis Using Trypanocides or Insecticide-Treated Livestock
Source: PLoS Negl Trop Dis. 2012 May 15;6(5):e1615. doi: 10.1371/journal.pntd.0001615 (PMC3352824; doi:10.1371/journal.pntd.0001615)
Supplement: Text S1 — Formulation of R 0. (DOC) [file pntd.0001615.s001.doc]

**Modeling the Control of Trypanosomiasis using Trypanocides or Insecticide-Treated Livestock**

**Supporting Material: Text S1**

**Formulation of *R*_0_**

*Model assumptions*

We derive an expression for the basic reproductive number (*R*_0_), generalizing the Rogers model [1] to include *n* host species. We assume that the population of each host species *i* = 1, 2,… , *n* is divided into three compartments, namely, susceptible ($S_{i}$), infective ($I_{i}$) and recovered ($R_{i}$). The vector population is assumed to comprise just two compartments, susceptible ($S_{V}$) and infective ($I_{V}$).

Susceptible species are recruited at rate$B_{i}$; they either die from natural causes, at rate$\mu_{i}$, or get infected at rate$\beta_{i}$. Infected species either die, at rate$\sigma_{i}$, or recover at rate $\rho_{i}$ and move to the recovered class. Recovered individuals may lose their immunity and move to the susceptible class at a rate $\nu_{i}$ or die at rate$\mu_{i}$.

Susceptible tsetse are generated at a rate $B_{V}$; they may die from natural causes, at rate *u*, or become infected after a blood meal from any infective species *i* where the probability of blood meal from species *i* is given by *a_i_* and the probability of an infected fly bite causing infection in species *i* is equal to *b_i_*. Infected flies are assumed to become infectious provided they survive an incubation period of length *T* with probability of survival given by$e^{-uT}$. Susceptible flies thus become infectious at rate$\lambda=e^{-uT}D\sum_{i=1}^{N} c_{i}a_{i}\frac{I_{i}(t-T)}{N_{i}(t-T)}$. Infectious flies are assumed to die, from natural causes only, at rate *u*.

Flowchart for the multi-host, single vector, model for trypanosomiasis transmission

*Mathematical model*

The system’s equations described by the flow chart are

$\begin{matrix} \frac{d}{dt}S_{i}=B_{i}+\nu_{i}R_{i}-\mu_{i}S_{i}-a_{i}b_{i}\frac{I_{V}}{N_{i}}S_{i} \\ \frac{d}{dt}I_{i}=a_{i}b_{i}\frac{I_{V}}{N_{i}}S_{i}-\rho_{i}I_{i}-\sigma_{i}I_{i} \\ \frac{d}{dt}R_{i}=\rho_{i}I_{i}-\mu_{i}R_{i}-\nu_{i}S_{i} \\ \frac{d}{dt}S_{V}=B_{V}-e^{-uT}D\sum_{i=1}^{N} c_{i}a_{i}\frac{I_{i}(t-T)}{N_{i}(t-T)}S_{V}(t-T)-uS_{V} \\ \frac{d}{dt}I_{V}=e^{-uT}D\sum_{i=1}^{N} c_{i}a_{i}\frac{I_{i}(t-T)}{N_{i}(t-T)}S_{V}(t-T)-uI_{V} \end{matrix}$ (A)

where $N_{i}=S_{i}+I_{i}+R_{i}$,

*D* = 1 for *T. vivax* and *T. congolense,* and

$D=\frac{u(1-\exp(-(a_{1}+a_{1}+a_{1}+....a_{i}+u))}{(a_{1}+a_{1}+a_{1}+....a_{i}+u)}$ for *T. brucei.*

*Basic reproductive number*

We derive the expression of the basic reproduction number using the next generation method developed in [1]. System (A) has a disease free equilibrium point $E_{0}=\left(\frac{B_{1}}{\mu_{1}},\cdots,\frac{B_{n}}{\mu_{n}},0,\cdots0,\frac{B_{V}}{u},0\right)$

Denote by **F** the vector formed by the model’s rates of appearance of new infections and by **V** the vector formed by the rates of the other transfers between compartments. We have **F** =$\left(\begin{matrix} a_{1}b_{1}\frac{I_{V}}{N_{1}}S_{1} \\ \vdots\\ a_{n}b_{n}\frac{I_{V}}{N_{n}}S_{n} \\ e^{-uT}D\sum_{i=1}^{N} c_{i}a_{i}\frac{I_{i}}{N_{i}}S_{V} \end{matrix}\right)$ **V** =$\left(\begin{matrix} (\sigma_{1}+\rho_{1})I_{1} \\ \vdots\\ (\sigma_{n}+\rho_{n})I_{n} \\ uI_{V} \end{matrix}\right)$.

The basic reproductive number is the spectral radius of F$V^{-1}$ where F and V are the Jacobian matrices at $E_{0}$ of **F** and **V** respectively. The eigenvalues of F$V^{-1}$ are given by the roots of the characteristic polynomial

$$P_{n+1}(z)=\det\left[\begin{matrix} -z & 0 & \cdots& 0 & \frac{a_{1}b_{1}}{u} \\ 0 & -z & \ddots& \vdots& \frac{a_{2}b_{2}}{u} \\ \vdots& \ddots& \ddots& 0 & \vdots\\ 0 & \cdots& 0 & -z & \frac{a_{n}b_{n}}{u} \\ e^{-uT}D\frac{a_{1}c_{1}m_{1}}{r_{1}} & e^{-uT}D\frac{a_{2}c_{2}m_{2}}{r_{2}} & \cdots& e^{-uT}D\frac{a_{n}c_{n}m_{n}}{r_{n}} & -z \end{matrix}\right]$$

where $r_{i}=\sigma_{i}+\rho_{i}$ (1/*r_i_* is thus the duration of infection in species *i*) and

$m_{i}=\frac{B_{V}/\mu_{V}}{B_{i}/\mu_{i}}$is the ratio of tsetse flies to host species *i*.

One can prove that

$P_{n+1}(z)=-zP_{n}(z)-(-z)^{n-1}\frac{e^{-uT}}{u}D\frac{a_{n}^{2}c_{n}b_{n}m_{n}}{r_{n}}$ implying that

$P_{n+1}(z)=(-z)^{n-1}\left(z^{2}-\frac{e^{-uT}}{u}D\sum_{i=1}^{n} \frac{a_{i}^{2}c_{i}b_{i}m_{i}}{r_{i}}\right)$.
According to [2], the basic reproductive number is the spectral radius of F$V^{-1}$., that is$\sqrt{\frac{e^{-uT}}{u}D\sum_{i=1}^{n} \frac{a_{i}^{2}c_{i}b_{i}m_{i}}{r_{i}}}$. This is “*the expected number of secondary cases produced, in a completely susceptible population, by a typical infective individual*” [3]. This definition does not distinguish between hosts and vectors in defining the “*typical infective individual*” and in the number of secondary cases. This explains the presence of the square root. In fact, two events are required to transmit an infection from fly to fly: first an infected tsetse fly must infect a host animal, then an uninfected fly must be infected when it bites the infected host.

Hence, we consider the “fly-to-fly” basic reproductive number which is the square of the basic reproductive number defined above. Finally, we assume that the$c_{i}$are all equal to a constant c. Thus:

$$R_{0}=\frac{c\exp(-uT)}{u}D\left(\frac{a_{1}^{2}b_{1}m_{1}}{r_{1}}+\frac{a_{2}^{2}b_{2}m_{2}}{r_{2}}+\frac{a_{3}^{2}b_{3}m_{3}}{r_{3}}+......\frac{a_{n}^{2}b_{n}m_{n}}{r_{n}}\right).$$

**References**

[1] Rogers DJ (1988) A general model for the African trypanosomiases. Parasitology 97: 193-212.

[2] Van den Driessche P, Watmough J (2002) Reproduction numbers and sub-threshold endemic equilibria for compartmental models of disease transmission. Mathematical Bioscience 180: 29–48.

[3] Diekmann O, Heesterbeek JAP, Metz JAJ (1990) On the definition and the computation of the basic reproduction ratio R0 in models for infectious diseases in heterogeneous populations. Journal of Mathematical Biology 28: 365–382.
